# The Effect of Personalized Oral Health Education on Oral Hygiene Behavior and Periodontal Health: A Meta‐Analysis and Systematic Review

**DOI:** 10.1155/ijod/7955459

**Published:** 2026-04-27

**Authors:** Yun Cai, Liping Wang, Raja Azman Raja Awang

**Affiliations:** ^1^ School of Dental Sciences, Health Campus, Universiti Sains Malaysia, Kubang Kerian, 16150, Kelantan, Malaysia, usm.my; ^2^ Key Laboratory of Oral Medicine, Guangzhou Institute of Oral Disease, Stomatology Hospital of Guangzhou Medical University, Guangzhou, Guangdong, China, gzhmc.edu.cn

**Keywords:** meta-analysis, periodontal health, personalized oral health education, systematic review

## Abstract

**Aims:**

The benefits of personalized oral health education on periodontal health are still controversial. This study aims to systematically evaluate the impact of personalized oral health education on periodontal health in the general population through meta‐analysis, and to provide evidence‐based guidance for governments, medical institutions, and healthcare workers on implementing primary prevention of periodontal problems.

**Methods:**

We conducted a search of Chinese and English databases to identify relevant literature published from the inception of each database to December 31, 2023, regarding the impact of personalized oral health education on periodontal health in the general population. Randomized controlled trials that met our inclusion and exclusion criteria were collected, and a quality assessment of the literature was performed. The risk of bias was assessed using the Cochrane risk of bias tool Version 2.0. The meta‐analysis was conducted using RevMan 5.4 and Stata 18.0 after the literature quality evaluation.

**Results:**

The search yielded 2690 articles. After rigorous screening, a total of eight papers were included, involving 3384 participants. The meta‐analysis showed that personalized oral health education, compared with conventional methods, significantly decreased plaque index (PI) (mean difference [MD] = −0.42, 95% confidence interval [CI; −0.63, −0.21], *p* < 0.0001) and gingival index (GI) (MD = −0.47, 95% CI [−0.67, −0.27], *p* < 0.0001). It also enhanced self‐efficacy and oral health‐related quality of life. Moreover, it increased the frequency of toothbrushing and dental flossing, and improved oral health knowledge, attitude, and behavior among participants.

**Conclusion:**

This study suggests that personalized oral health education can significantly improve periodontal health.

## 1. Introduction

Periodontitis is the sixth most common disease worldwide [1]. According to the Global Burden of Disease report in 2017, the prevalence of severe periodontitis is estimated to be around 11%, affecting approximately 743 million people [2]. The prevalence of mild periodontitis is even higher, affecting at least half of the global population [3]. The incidence of periodontitis increases with age, reaching its peak between the ages of 50 and 60 [4]. The severity of the disease also increases the complexity of treatment and the associated costs [5]. Improving periodontal health and implementing the three levels of prevention for periodontal disease are an absolute obligation for the government, medical education institutions, and healthcare professionals [6, 7]. The primary prevention targets the entire population, with a focus on children and adolescents [8]. To prevent the harmful effects of plaque and other pathogenic factors on healthy periodontal tissues, it is important to remove these factors before they cause damage [9]. This can be achieved through oral hygiene education and regular dental check‐ups, which help individuals establish good personal oral hygiene habits and learn the correct brushing techniques [10]. Controlling dental plaque can prevent the occurrence of gingivitis [11]. Goodarzi et al. [12] found that families with low levels of education do not prioritize oral health prevention. Ludovichetti et al. [13] found that caregivers lack oral health knowledge, and Dumitrescu et al. [14] found that the majority of people have not received oral health education. These findings highlight the urgent need for oral health education and awareness promotion.

Additionally, related studies have also indicated that traditional oral health education has been ineffective [15–17]. Harnacke et al. [18] conducted a study involving 83 students without clinical symptoms of periodontitis. The participants were randomly assigned to a control group or three experimental groups: (1) written education, (2) standardized oral education, and (3) personalized oral health education. Plaque index (PI) and bleeding index were assessed at baseline and 4 weeks after the intervention. The results showed significant differences among the groups in terms of gingival bleeding, with significant temporary differences observed in oral hygiene skills. Participants who received personalized oral health education demonstrated the best outcomes [18]. In another study, Almabadi et al. [19] divided 295 participants with an average age of 45.4 ± 11 years into two groups. The experimental group received dental treatment along with a personalized oral health education program. The results showed that personalized oral health education had beneficial effects [19]. In a randomized controlled clinical trial conducted by Gunpinar and Meraci [20], 50 patients with gingivitis who were undergoing periodontal treatment were studied. The participants were randomly divided into the personalized health education session (PHES) group (experimental group) and the standard oral health education group (control group). PI and gingival bleeding index were used to assess the baseline oral hygiene status and were reevaluated at 1, 3, and 6 months. The study showed that although there were no differences in baseline knowledge levels and motivation scores between the two groups, the experimental group exhibited higher levels of improvement in oral health after receiving personalized sessions [20]. Indeed, increasing the understanding and awareness of periodontal disease and its consequences among individuals with periodontitis can improve their oral hygiene. However, there is still controversy over whether oral health education for other populations (not just those with periodontitis) can improve periodontal health. In a 2‐year randomized intervention study conducted by Komulainen et al. [21], 279 community‐dwelling elderly individuals were divided into two groups. Personalized oral health education included individualized guidance on oral and/or denture hygiene, alleviating dry mouth symptoms, reducing the frequency of sugar use, using fluoride, xylitol, or antimicrobial products, and professional teeth cleaning. The results showed that compared to baseline, a greater number of participants in the intervention and control groups had improved oral and denture hygiene, and no oral diseases or symptoms were observed at the 2‐year follow‐up. However, the differences in outcome changes between the intervention and control groups were not statistically significant [21]. In a study conducted by Donos et al. [22], 71 participants were randomly assigned to receive personalized oral health education or traditional oral health education. The results showed that, compared with the standard approach, personalized oral health education based on two visits did not provide additional benefits [22]. Therefore, the question of whether to promote personalized oral health education to all populations, not just periodontal disease patients, remains unclear. More research and empirical data accumulation will contribute to a deeper understanding of the universality and practical effectiveness of personalized oral health education, enabling a more comprehensive evaluation of its applicability in different populations.

This study aims to evaluate, through a meta‐analysis, the impact of personalized oral health education on periodontal health across diverse populations, in order to provide evidence‐based support for the government agencies, medical educators, and healthcare professionals in implementing primary prevention strategies.

## 2. Methods

This protocol adhered to the Preferred Reporting Items for Systematic Reviews and Meta‐Analyses 2020 (PRISMA 2020) statement and followed the five‐stage approach described elsewhere. The completed PRISMA 2020 checklist can be found in Supporting Information.

### 2.1. Literature Search

We searched both Chinese and English databases using a combination of keywords and free‐text terms. We collected studies on the impact of oral health education on oral hygiene behavior and periodontal health that were published up to December 31, 2023. The English databases searched included PubMed, Embase, the Cochrane Library, EBSCO, Scopus, and Web of Science. The Chinese databases searched were CNKI, Wanfang, and VIP. The core search strategy was as follows: (“Health Education” [MeSH] OR “Education, Health” [All Fields] OR “Community Health Education” [All Fields] OR “Education, Community Health” [All Fields] OR “Health Education, Community” [All Fields]) AND (“Periodontal Health” [All Fields]) AND (“Randomized Controlled Trial” [All Fields]). The complete search strategy is available in Table S1.

### 2.2. Inclusion/Exclusion Criteria

#### 2.2.1. Inclusion Criteria

Studies were included if they met the following criteria: (1) the study design was a randomized controlled trial; (2) the experimental group consisted of periodontal patients or healthy subjects who received personalized oral health education; and (3) the control group consisted of periodontal patients or healthy subjects who did not receive personalized oral health education.

#### 2.2.2. Exclusion Criteria

Studies were excluded if they met any of the following criteria: (1) non‐randomized controlled trials, in vitro studies, or animal studies; (2) studies including subjects with systemic diseases such as diabetes or cardiovascular disease; (3) studies involving participants who had taken antibiotics or other medications affecting periodontal tissue before or after the intervention, or who received antibiotics or surgical treatment during the follow‐up period; (4) studies lacking outcome indicators, or failing to provide sufficient data to calculate weighted mean difference (WMD), risk ratio (RR), and 95% confidence intervals (CIs); (5) duplicate publications or studies with incomplete data; or (6) studies published as lecture notes, conference abstracts, reports, dissertations, or reviews.

### 2.3. Outcome Measures

The primary outcome measures are the PI, gingival index (GI), frequency of brushing, and oral hygiene knowledge. The secondary outcome measures include self‐efficacy, oral health‐related quality of life, frequency of dental floss usage, oral hygiene attitude, and oral hygiene practice.

### 2.4. Data Extraction

The literature search and study selection were conducted by two researchers who independently screened the articles. After the searches were performed in electronic databases, all identified articles were imported into reference management software (EndNote X9 and Microsoft Excel). Duplicate articles were removed. The titles and abstracts were examined to identify articles that did not meet the inclusion criteria. Finally, the full texts of the remaining articles were retrieved and assessed for eligibility. Articles that met all inclusion criteria were selected. Any discrepancies in the literature selection process were resolved through consultation with relevant experts or discussion with a third researcher. If multiple articles were published based on the same study, the most comprehensive and up‐to‐date article was included. Microsoft Excel was used to organize and extract data from all eligible literature, including (1) basic information (authors, country, publication year); (2) participant characteristics (age, gender, and sample size); (3) study design (intervention measures, follow‐up duration); and (4) outcome measures. In instances where the units of the outcome measures varied across the literature, they were uniformly converted before data processing. If the literature lacked certain data, the original authors were contacted through email or phone to collect the necessary information.

### 2.5. Literature Quality Assessment

The Cochrane Risk of Bias tool version 2.0 was employed to assess the methodological quality of the included articles. The tool covers five domains: the randomization process, deviations from intended interventions, missing outcome data, measurement of the outcome, and selection of the reported result. Each domain was rated as “low risk of bias,” “some concerns,” or “high risk of bias.” An article was classified as having an overall judgment of: (1) “low risk of bias” if all domains were rated as low risk; (2) “some concerns” if at least one domain was rated as some concerns, but no domain was rated as high risk; and (3) “high risk of bias” if at least one domain was rated as high risk. Any disagreements during the appraisal process were resolved through discussion with a third reviewer.

### 2.6. Statistical Analysis

Meta‐analysis was conducted using the Cochrane Collaboration’s RevMan 5.4 software. For the analysis of continuous variables, the mean difference (MD) and 95% CI were used, and a forest plot was generated. In this study, the difference between post‐intervention and baseline values was utilized as the effect size parameter. The following formulas were used:

S2=S12+212−2×R×S1×S2,

M=M1−M2,

where *S* is the standard deviation of the effect, *S*_1_ is the baseline standard deviation, *S*_2_ is the post‐intervention standard deviation, *R* is the constant 0.4 or 0.5, *M* is the mean effect, *M*_1_ is the baseline mean, and *M*_2_ is the post‐intervention mean.

The *I* ^2^ statistic was used to quantify heterogeneity, and if *I* ^2^ < 50%, the heterogeneity was considered acceptable, and a fixed‐effect model was employed for the meta‐analysis. If *I* ^2^ ≥ 50%, indicating substantial statistical heterogeneity, sensitivity analysis and subgroup analysis were conducted. When the source of heterogeneity could not be explained by clinical or methodological heterogeneity, a random‐effects model was used to combine the effect sizes. If the heterogeneity was too substantial, a descriptive statistical analysis was performed. The Stata 18.0 software was used to conduct Egger’s test and Begg’s test to assess publication bias. A *p*‐value of < 0.05 in Egger’s test or Begg’s test was considered significant publication bias.

## 3. Results

### 3.1. Search Results and Procedure

**Figure 1:**
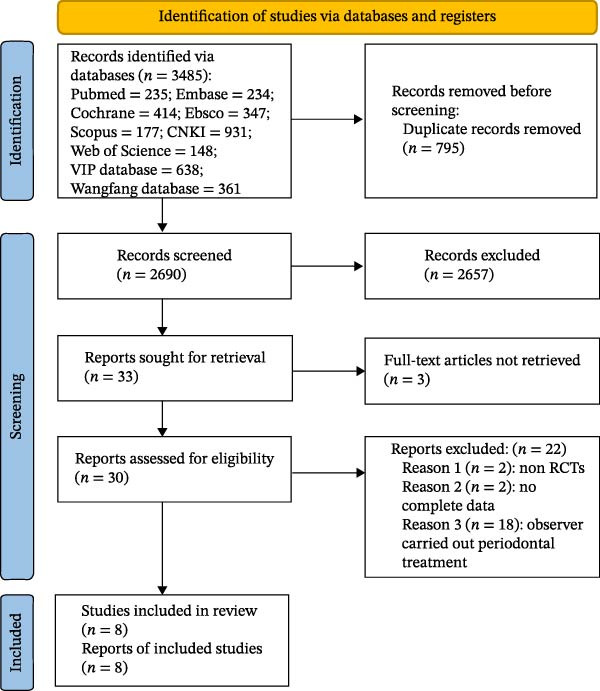
Flowchart of the literature screening process.

The process of study identification and selection is presented in the PRISMA flow diagram (Figure 1). A total of 2690 records were identified through database searches up to December 2023. After duplicates were removed, two researchers independently screened the titles and abstracts against the predefined inclusion and exclusion criteria. They cross‐checked the results and identified records requiring full‐text retrieval. A total of 2657 records were excluded during title and abstract screening, leaving 33 records for full‐text assessment. Of these, three records could not be retrieved in full text, resulting in 30 records proceeding to full‐text review. During full‐text review, a further 22 records were excluded for the following reasons: not a randomized controlled trial (*n* = 2), incomplete data (*n* = 2), and participants receiving periodontal treatment (*n* = 18). Ultimately, eight studies were included in this review [23–30].

### 3.2. Basic Characteristics of Included Literature

The eight included studies were all randomized controlled trials. These studies were conducted between 1985 and 2023 and were published in Europe (*n* = 4), Asia (*n* = 2), and America (*n* = 2). The control group in these trials received conventional oral health education. The outcome measures included: (1) PI, (2) GI, (3) self‐efficacy, (4) oral health‐related quality of life, (5) frequency of brushing, (6) frequency of dental floss usage, (7) oral hygiene knowledge, (8) oral hygiene attitude, and (9) oral hygiene practice. Refer to Table 1 for specific details.

**Table 1:** Basic characteristics of included literature.

Author/year (continent)	Age (year)	Gender (male/female)	Sample size	Intervention	Follow‐up (mouth)	Outcome
D’Cruz and Aradhya [25] (Asia)	Control group: 13–15 Experimental group: 13–15	Control group: 147/137 Experimental group: ⑴ 67/74 ⑵ 68/75	Control group: 284 Experimental group: ⑴ 141 ⑵ 143	Control group: regular oral health education Experimental group: ⑴ lecture using a PowerPoint presentation ⑵ lecture using a PowerPoint presentation with toothbrushing demonstration	9	① ② ⑦ ⑨
Al Khamis et al. [28] (Europe)	Control group: 28.8 ± 5.7 Experimental group: ⑴ 27.7 ± 5.6 ⑵ 26.97 ± 4.8	Control group: 0/28 Experimental group: ⑴ 0/30 ⑵ 0/32	Control group: 28 Experimental group: ⑴ 30 ⑵ 32	Control group: regular oral health education Experimental group: ⑴ Dental hygienist education (DHE) ⑵ Dental hygienist education and planning (DHE&P)	1	① ② ⑤ ⑥ ⑦ ⑧
Walsh [24] (America)	Control group: 12–14 Experimental group: 12–14	—	Control group: 240 Experimental group: 399	Control group: regular oral health education Experimental group：a series of classroom preventive dentistry presentations on dental health knowledge, attitudes, and reported behavior	—	⑦ ⑧
Saied‐Moallemi et al. [26] (Europe)	Control group: 9 Experimental group: 9	224/233	Control group: 116 Experimental group: ⑴ 110 ⑵ 112 ⑶ 109	Control group: regular oral health education Experimental group: ⑴ Class‐work ⑵ Parental‐aid ⑶ Combined	3	① ②
Julien [27] (America)	Control group: 10 Experimental group: 10	Control group: 104/93 Experimental group: 109/97	Control group: 153 Experimental group: 163	Control group: regular oral health education Experimental group: a programme based on the use of several behavior modification strategies	15	① ②
Pakpour et al. [29] (Europe)	Control group: 15.37 ± 1.32 Experimental group: ⑴ 15.43 ± 1.50 ⑵ 15.26 ± 1.33	Control group: 200/185 Experimental group: ⑴ 188/198 ⑵ 186/201	Control group: 385 Experimental group: ⑴ 386 ⑵ 387	Control group: regular oral health education Experimental group: ⑴ Implementation intention ⑵ Action planning	—	① ③ ④ ⑤
Gholami M et al. [23] (Europe)	Control group: 12.5 ± 1.14 Experimental group: 12.5 ± 1.14	—	Control group: 97 Experimental group: 69	Control group: regular oral health education Experimental group: a brief self‐regulatory intervention	1	③ ⑥
Pai Khot et al. [30] (Asia)	Control group: 7–18 Experimental group: 7–18	38/22	Control group: 30 Experimental group: 30	Control group: conventional verbal techniques for Oral Health Education (OHE) Experimental group: picture‐assisted illustration reinforcement (PAIR) communication system	3	⑨

### 3.3. Quality Assessment of Literature

**Figure 2:**
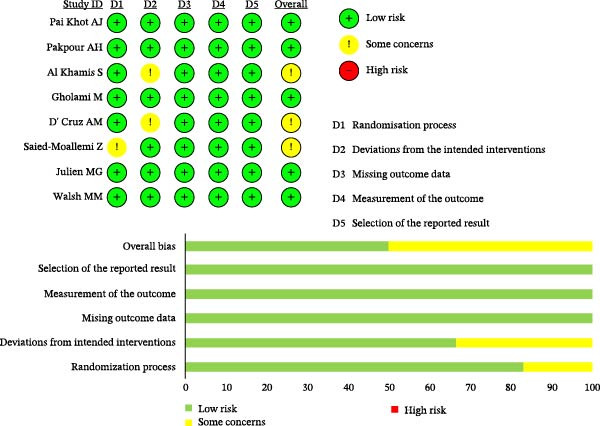
Risk of bias graph of the included studies.

The quality of the eight included studies was evaluated using the Cochrane Risk of Bias tool, with results presented in Figure 2. Regarding the randomization process, all studies were rated as low risk of bias except for Pai Khot et al. [30] which was judged as “some concerns” due to an unclear description of the randomization strategy [26]. For deviations from intended interventions, two studies were rated as “some concerns” because the intervention implementers could not be blinded, while the remaining six studies were rated as low risk of bias [25, 28]. In the domains of missing outcome data, outcome measurement, and selective reporting, all eight studies were rated as low risk of bias. Overall, the included studies demonstrated good methodological quality.

### 3.4. Meta‐Analysis Outcomes

This study included a total of eight articles and involved 3384 participants. According to the intervention format, the personalized oral health education methods adopted in the included studies can be classified into four categories: (1) individualized face‐to‐face guidance, including toothbrushing demonstrations combined with PowerPoint presentations, as well as one‐on‐one counseling focusing on action planning, self‐efficacy, and behavioral intention cultivation; (2) group‐based interventions, encompassing classroom‐based group interventions and combined group sessions integrating multiple activities; (3) support systems, primarily referring to parent support groups that target parents to indirectly influence patients; and (4) mixed‐mode interventions, namely comprehensive interventions combining PowerPoint visual presentations with hands‐on toothbrushing demonstration training. Subsequently, data extraction was conducted separately for each educational method, and subgroup analyses were performed for different follow‐up times.

#### 3.4.1. PI

Five studies examined the effect of oral health education on plaque control, comprising a total of 3344 participants, with 1523 in the intervention group and 1821 in the control group. The overall heterogeneity test revealed an *I* ^2^ value of 95%; therefore, a random‐effects model was used for the analysis (Figure 3a). The overall meta‐analysis revealed a MD of −0.42 (95% CI: −0.63 to −0.21), indicating a statistically significant difference (*p* < 0.0001). Due to the substantial heterogeneity observed across the included studies (*I* ^2^ = 95%), subgroup analyses were conducted to explore potential sources of heterogeneity. Subgroup analysis based on follow‐up time substantially reduced heterogeneity (Figure S1). The results indicated that the persistence of the intervention effect was time‐dependent: at 1 month post‐education, the meta‐analysis showed an MD of −0.26 (95% CI: −0.52–0.01), with heterogeneity (*I* ^2^ = 87%), indicating no statistically significant difference between the two groups at this time point (*p* = 0.06). At 3 months post‐education, the meta‐analysis revealed an MD of −0.27 (95% CI: −0.32 to −0.23), with no heterogeneity (*I* ^2^ = 0%), indicating that the oral health education intervention was significantly more effective than the control condition in reducing plaque levels (*p* < 0.00001). At 6 months post‐education, the meta‐analysis showed an MD of −0.38 (95% CI: −0.49 to −0.28), with heterogeneity (*I* ^2^ = 77%), indicating a significantly lower PI in the intervention group compared to the control group (*p* < 0.00001). Furthermore, at 9 months post‐education, the meta‐analysis revealed an MD of −0.64 (95% CI: −0.86 to −0.42), with heterogeneity (*I* ^2^ = 96%), indicating a statistically significant beneficial effect of oral health education on reducing PI (*p* < 0.00001).

Figure 3Meta‐analysis forest plot assessing the influence of oral health education on the population’s (a) plaque index, (b) gingival index, (c) self‐efficacy, and (d) oral health‐related quality of life.

**Figure:**
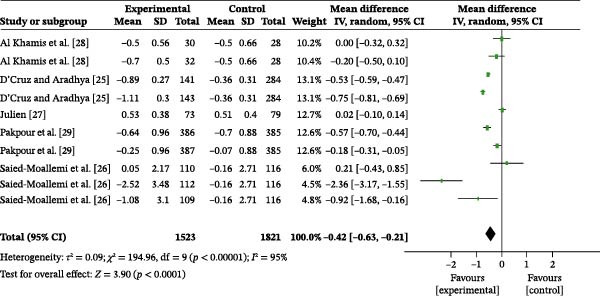
(a)

**Figure:**
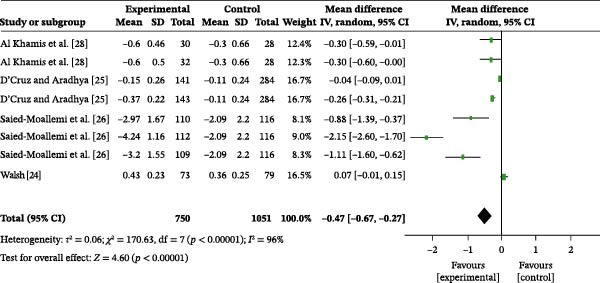
(b)

**Figure:**
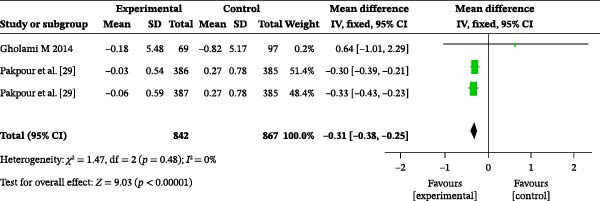
(c)

**Figure:**
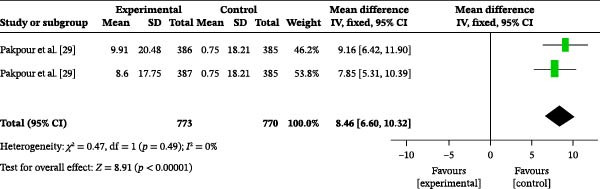
(d)

#### 3.4.2. GI

Four studies examined the effect of oral health education on gingival bleeding control, comprising a total of 1801 participants, with 750 in the intervention group and 1051 in the control group. The overall heterogeneity test revealed an *I* ^2^ value of 96%; therefore, a random‐effects model was used for the analysis (Figure 3b). The overall meta‐analysis revealed a MD of −0.47 (95% CI: −0.67 to −0.27), indicating a statistically significant difference (*p* < 0.00001). Due to the substantial heterogeneity observed across the included studies (*I* ^2^ = 96%), subgroup analyses were conducted to explore potential sources of heterogeneity. Subgroup analysis based on follow‐up time substantially reduced heterogeneity (Figure S2). The results indicated that the persistence of the intervention effect was time‐dependent: at 1 month post‐education, the meta‐analysis showed an MD of −0.30 (95% CI: −0.51 to −0.09), with no heterogeneity (*I* ^2^ = 0%), indicating that oral health education was beneficial in reducing GI (*p* = 0.005). At 3 months post‐education, the meta‐analysis revealed an MD of −0.04 (95% CI: −0.07 to −0.001), with no heterogeneity (*I* ^2^ = 0%), indicating that the intervention group exhibited significantly better control of gingival bleeding compared to the control group (*p* = 0.04). Similarly, at 6 months post‐education, the meta‐analysis showed an MD of −0.08 (95% CI: −0.11 to −0.05), with no heterogeneity (*I* ^2^ = 0%), indicating a significantly lower GI in the intervention group compared to the control group (*p* < 0.00001). However, at 9 months post‐education, the meta‐analysis revealed an MD of −0.15 (95% CI: −0.37–0.07), with heterogeneity (*I* ^2^ = 97%), indicating no statistically significant difference between the two groups at this time point (*p* = 0.17).

#### 3.4.3. Self‐Efficacy

Two studies reported on the impact of oral health education on self‐efficacy. A total of 1709 participants were included in these studies, with 842 in the intervention group and 867 in the control group. The overall heterogeneity test showed an *I* ^2^ value of 0% (Figure 3c); therefore, a fixed‐effects model was used for the analysis. The overall meta‐analysis revealed a MD of −0.31 (95% CI: −0.38 to −0.25), indicating a statistically significant difference (*p* < 0.00001). Subgroup analysis based on follow‐up time was performed. At 1 month post‐education, there was no significant difference in self‐efficacy between the two groups (MD = 0.15, 95% CI: −0.05–0.35, *p* = 0.15) (Figure S3). However, at 6 months post‐education, there was a significant difference in self‐efficacy between the two groups (MD = −0.31, 95% CI: −0.38 to −0.25, *p* < 0.00001), indicating that self‐efficacy was significantly higher in the intervention group than in the control group.

#### 3.4.4. Oral Health‐Related Quality of Life

One study reported on the impact of oral health education on oral health‐related quality of life. A total of 1543 participants were included, with 773 in the intervention group and 770 in the control group. The heterogeneity test showed an *I* ^2^ value of 0% (Figure 3d); therefore, a fixed‐effects model was used for the analysis. The meta‐analysis revealed a MD of 8.46 (95% CI: 6.60–10.32), indicating that oral health education significantly improved oral health‐related quality of life (*p* < 0.00001).

#### 3.4.5. Frequency of Brushing

Two studies reported the influence of oral health education on brushing frequency. A total of 1661 participants were included, with 835 in the intervention group and 826 in the control group. The overall heterogeneity test showed an *I* ^2^ value of 90% (Figure 4a); therefore, a random‐effects model was used for the analysis. The overall meta‐analysis revealed a MD of 1.15 (95% CI: 0.09–2.21), indicating a statistically significant difference (*p* = 0.03). Due to the substantial heterogeneity observed across the included studies *(I^2^* = 90%), subgroup analyses were conducted to explore potential sources of heterogeneity. Subgroup analysis based on follow‐up time reduced heterogeneity (Figure S4). The results indicated that the persistence of the intervention effect was time‐dependent: At 1 month post‐education, the meta‐analysis showed an MD of 1.17 (95% CI: 0.07–2.28), with heterogeneity (*I* ^2^ = 91%), indicating that oral health education was superior to the control condition in improving brushing frequency (*p* = 0.04). At 6 months post‐education, the meta‐analysis showed an MD of 2.26 (95% CI: 1.02–3.49), with heterogeneity (*I* ^2^ = 69%), indicating that oral health education significantly increased brushing frequency (*p* = 0.0003).

Figure 4Meta‐analysis forest plot of the impact of oral health education on the frequency of (a) brushing and (b) dental floss usage in the population.

**Figure:**
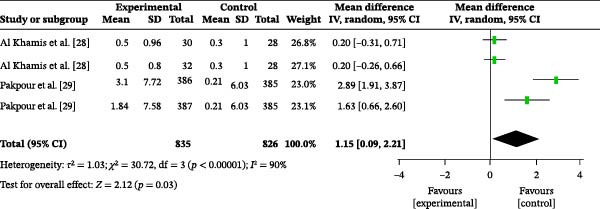
(a)

**Figure:**
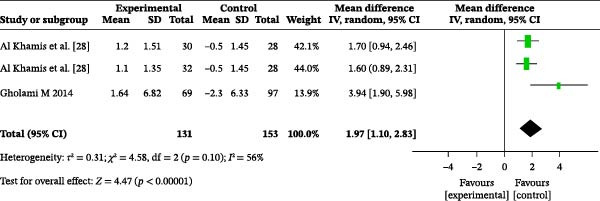
(b)

#### 3.4.6. Frequency of Dental Floss Usage

Two studies reported the impact of oral health education on dental floss usage frequency. A total of 284 participants were included, with 131 in the intervention group and 153 in the control group. The heterogeneity test showed an *I* ^2^ value of 56% (Figure 4b); therefore, a random‐effects model was used for the analysis. The meta‐analysis revealed a MD of 1.97 (95% CI: 1.10–2.83), indicating that oral health education significantly increased dental floss usage frequency (*p* < 0.00001).

#### 3.4.7. Oral Hygiene Knowledge

Three studies reported the impact of oral health education on oral hygiene knowledge. A total of 1609 participants were included, with 745 in the intervention group and 864 in the control group. The overall heterogeneity test showed an *I* ^2^ value of 99% (Figure 5a); therefore, a random‐effects model was used for the analysis. The overall meta‐analysis revealed a MD of 2.14 (95% CI: 0.59–3.69), indicating a statistically significant difference (*p* = 0.007). Due to the substantial heterogeneity observed across the included studies (*I* ^2^ = 99%), subgroup analyses were conducted to explore potential sources of heterogeneity. Subgroup analysis based on follow‐up time reduced heterogeneity (Figure S5). The results indicated that the persistence of the intervention effect was time‐dependent: at 1 month post‐education, the meta‐analysis showed an MD of 0.70 (95% CI: −0.20 to 1.59), with no heterogeneity (*I* ^2^ = 0%), indicating no significant difference in oral hygiene knowledge between the two groups (*p* = 0.13). However, at 6 months post‐education, the meta‐analysis showed an MD of 4.05 (95% CI: 3.58–4.52), with heterogeneity (*I* ^2^ = 81%), indicating that oral hygiene knowledge was significantly higher in the intervention group than in the control group (*p* < 0.00001). At 9 months post‐education, the meta‐analysis showed an MD of 3.94 (95% CI: 3.55–4.34), with heterogeneity (*I* ^2^ = 68%), indicating that oral health education significantly improved oral hygiene knowledge (*p* < 0.00001).

Figure 5Meta‐analysis forest plot assessing the influence of oral health education on the population’s oral hygiene (a) knowledge, (b) attitude, and (c) practices.

**Figure:**
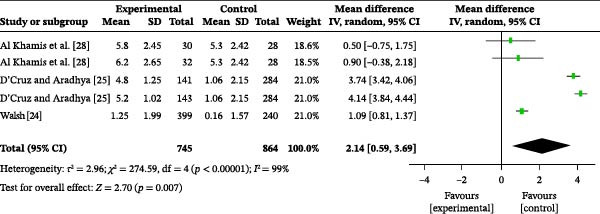
(a)

**Figure:**
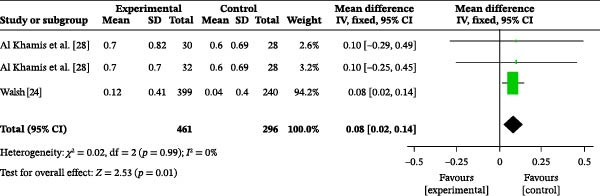
(b)

**Figure:**
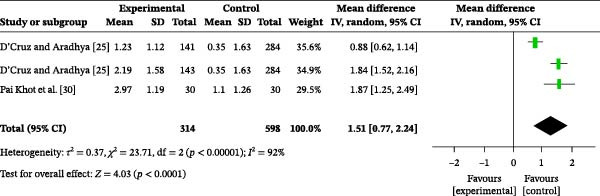
(c)

#### 3.4.8. Oral Hygiene Attitude

Two studies reported the impact of oral health education on oral health attitudes. A total of 757 participants were included, with 461 in the intervention group and 296 in the control group. The heterogeneity test showed an *I* ^2^ value of 0% (Figure 5b); therefore, a fixed‐effects model was used for the analysis. The meta‐analysis revealed a MD of 0.08 (95% CI: 0.02–0.14), indicating that oral health education significantly improved oral health attitudes (*p* = 0.01).

#### 3.4.9. Oral Hygiene Practice

One study reported the impact of oral health education on oral hygiene practices. A total of 912 participants were included, with 314 in the intervention group and 598 in the control group. The overall heterogeneity test showed an *I* ^2^ value of 92% (Figure 5c); therefore, a random‐effects model was used for the analysis. The overall meta‐analysis revealed a MD of 1.51 (95% CI: 0.77–2.24), indicating a statistically significant difference (*p* < 0.00001). Subgroup analysis based on follow‐up time was performed. At 3 months post‐education, there was no significant difference in oral hygiene practices between the two groups (MD = 0.52, 95% CI: −1.21–2.26, *p* = 0.55) (Figure S6). However, at 6 months post‐education, oral hygiene practices were significantly better in the intervention group than in the control group (MD = 1.14, 95% CI: 0.01–2.27, *p* = 0.05). Furthermore, at 9 months post‐education, oral health education significantly improved oral hygiene practices (MD = 1.36, 95% CI: 0.41–2.30, *p* = 0.005).

#### 3.4.10. Follow‐Up Times

The follow‐up periods ranged from 1 to 27 months. Following personalized oral health education, the PI showed no significant improvement at 1 month, but significant improvements were observed at 3, 6, and 9 months. The GI significantly improved at 1, 3, and 6 months, but the effect dissipated by 9 months. Self‐efficacy showed no improvement at 1 month but significantly increased at 6 months. Brushing frequency significantly improved at 1 and 6 months. Oral hygiene knowledge showed no improvement at 1 month but significantly improved at 6 and 9 months. Oral hygiene practices showed no improvement at 3 months but significantly improved at 6 and 9 months. One study reported that at 27 months of follow‐up, the PI in the intervention group remained lower than that in the control group; however, the difference was not sufficient to maintain a significant difference in the GI.

### 3.5. Publication Bias

Egger’s test and Begg’s test were performed simultaneously in this study, and the results indicated that the included literature did not show any significant publication bias, as shown in Table 2.

**Table 2:** Publication bias.

Indicators	Egger’s test	Begg’s test
Plaque index	0.120	0.764
Gingival index	0.939	0.806
Self‐efficacy	0.247	1.000
Tooth brushing	0.054	0.089
Oral hygiene practice	0.593	1.000

## 4. Discussion

This study suggested that oral health education was effective in improving oral health knowledge. Consequently, it could also improve oral health attitudes, behaviors, and self‐efficacy, ultimately enhancing oral health‐related quality of life. Furthermore, it might increase toothbrushing and dental floss frequency, thereby reducing plaque and gingival bleeding indices, and ultimately improving periodontal health. This was one of the first studies to evaluate the effectiveness of personalized oral health education on periodontal health. The research demonstrated that personalized oral health education could improve periodontal health, which is consistent with international studies [31–33]. Unlike previous studies that mainly focused on post‐treatment oral health education for chronic periodontitis patients, this study included individuals from the general population without prior periodontal treatment. Personalized oral health education is a structured learning activity involving information and experience sharing that promotes oral health in different target populations through various tailored approaches [19].

This study included a total of eight articles and involved a population of 3384 participants. D’Cruz and Aradhya [25] employed two personalized educational methods for the intervention group: PowerPoint presentations and PowerPoint presentations combined with toothbrushing demonstrations. Khamis et al. [28] used two personalized educational methods for the intervention group: oral health education with a focus on social cognition, and oral health education with a focus on social cognition combined with planning [28]. Julien [27] conducted an intensive training program for the intervention group over a 4‐month period, including dental examinations, regular visits by oral health workers to schools to teach toothbrushing and flossing techniques and monitor children’s performance, and signing a “contract” with the program coordinator and a parent for each child to encourage commitment and reinforce expected behaviors. The control group allowed students to receive oral hygiene education through personal contact with their chosen dentist [27]. Walsh [24] conducted four 1 h sessions of personalized oral health education for the intervention group. Saied‐Moallemi et al. [26] employed three personalized educational approaches for classroom groups, parent support groups, and a combined group. Pakpour et al. [29] implemented two personalized oral health education methods for the intervention and intention‐to‐treat groups. Gholami et al. [23] conducted personalized education on planning, self‐efficacy, and behavioral intentions for the intervention group. Pai Khot et al. [30] provided personalized education for the intervention group utilizing “picture‐assisted illustration reinforcement.” This study demonstrated that personalized oral health education had a positive impact, as there was a significant improvement in knowledge scores after educational intervention. This finding was consistent with several prior studies. For example, Biesbrock et al. [34] reported an 86% increase in knowledge scores (*p* < 0.001) following a 4‐week oral health education program. Walsh [24] reported a 44.8% change in knowledge levels in the experimental group, compared to a 5.4% change in the control group. Alsaadoon et al. [35] found a 95% increase in oral health knowledge after a dental storybook‐based oral health education program compared to other groups. The improvement in knowledge levels was necessary and a key determinant of attitude and behavior change. In addition, the research also showed that personalized oral health education has a positive impact on the attitudes, behaviors, and self‐efficacy of individuals towards oral health, leading to an improvement in their oral health‐related quality of life. This highlights that an increase in knowledge level is not only necessary but also a key factor in changing attitudes, behaviors, and self‐efficacy. Therefore, personalized oral health education holds potential importance in comprehensive oral health improvement, providing targeted educational programs for different populations. However, as the research progresses, further studies and exploration are needed to identify the optimal match between specific populations and educational approaches.

This study demonstrated that personalized oral health education improves brushing frequency and dental floss usage among the population. This might be attributed to the fact that personalized oral health education enhances oral health knowledge, thereby improving oral health behaviors. Consistent with other studies [36], this research also found that personalized oral health education is beneficial in reducing plaque scores and gingival bleeding scores among the population. This might be because gingival bleeding occurs as a result of plaque accumulation and maturation. Therefore, the improvement in brushing frequency and dental floss usage also led to a decrease in plaque scores and gingival bleeding scores. Maintaining oral cleanliness, especially ensuring that tooth surfaces are free from plaque, is the cornerstone of preventing periodontal diseases [37]. Good oral health behaviors could significantly reduce the occurrence of periodontal issues and other related problems [38]. This study found that follow‐up duration was one of the main sources of heterogeneity in the effects of oral health education. After stratification by follow‐up time, the heterogeneity within each subgroup was significantly reduced, and the pooled effect sizes of all subgroups were located on the same side of the line of no effect, further confirming the overall effectiveness of personalized oral health education while revealing the dynamic pattern of its effects over time. Clinicians and policymakers should recognize that a single session of health education may struggle to produce lasting effects. It is recommended to transform health education from a one‐time campaign into a sustained intervention model that includes long‐term follow‐up, reminders, and reinforcement, in order to maintain its effectiveness over extended follow‐up periods.

The limitations of this systematic review included the inclusion of only English‐language publications due to difficulties in assessing reports in other languages. While personalized oral health education might enhance oral health knowledge, ensuring the sustainability of knowledge remains a challenge. Several key areas need to be addressed to ensure the sustainability and implementation of practices, including ongoing continuing education and training programs to update the oral health knowledge of non‐dental healthcare professionals, as well as encouraging interdisciplinary collaboration to adopt a holistic approach to patient care. Long‐term studies are needed in the future to evaluate the sustainability of knowledge provided and examine various strategies that can facilitate its implementation.

## 5. Conclusion

### 5.1. Implications for Clinical Practice

Personalized oral health education may contribute to improving oral health knowledge among the general population, which could positively influence attitudes and behaviors related to oral hygiene. Tailored educational approaches may help individuals develop greater self‐efficacy, potentially supporting more consistent toothbrushing and flossing habits. These behavioral changes may, in turn, aid in maintaining periodontal health, reducing plaque accumulation, and minimizing gingival bleeding, thereby potentially enhancing patients’ oral health‐related quality of life.

### 5.2. Implications for Research

Further research would be valuable to investigate the long‐term effects of personalized oral health education across diverse populations. Studies could usefully examine effective educational methods and materials for different demographic groups and settings. Additionally, investigating the relationship between improved oral health knowledge and actual clinical outcomes may provide further insights into the efficacy of personalized interventions. Continued inquiry in this area may contribute to the development of evidence‐based practices that could be implemented in diverse clinical settings.

**Nomenclature**
MD: Mean difference
CI: Confidence interval
PI: Plaque index
GI: Gingival index.

## Author Contributions

**Yun Cai:** literature search, data extraction, formal analysis, writing – original draft, writing – review and editing. **Liping Wang:** literature search, data extraction. **Raja Azman Raja Awang:** conceptualization, resources, supervision.

## Funding

This study did not receive any funding support.

## Ethics Statement

The study protocols registered with PROSPERO, titled “The effect of personalized oral health education on oral hygiene behavior and periodontal health: A Meta‐analysis and system evaluation,” Number CRD42023421707.

## Conflicts of Interest

The authors declare no conflicts of interest.

## Supporting Information

Additional supporting information can be found online in the Supporting Information section.

## Supporting information


**Supporting Information** Both Supporting materials can be found in their respective sections of the manuscript. Figure S1. Subgroup analysis on the impact of oral health education on the population’s plaque index. Figure S2. Subgroup analysis on the impact of oral health education on the population’s gingival index. Figure S3. Subgroup analysis on the impact of oral health education on the population’s self‐efficacy. Figure S4. Subgroup analysis of the impact of oral health education on the frequency of brushing in the population. Figure S5. Subgroup analysis on the impact of oral health education on the population’s oral hygiene knowledge. Figure S6. Subgroup analysis on the impact of oral health education on the population’s oral hygiene practice. Table S1. Search strategy on Pubmed.

## Data Availability

The data that support the findings of this study are available from the corresponding author upon reasonable request.
