# Supplementary material for: The Effect of Personalized Oral Health Education on Oral Hygiene Behavior and Periodontal Health: A Meta‐Analysis and Systematic Review
Source: Int J Dent. 2026 Apr 27;2026:7955459. doi: 10.1155/ijod/7955459 (PMC13121865; doi:10.1155/ijod/7955459)

**Supplementary materials**

**Table legends**

Table S1: Search strategy on Pubmed.

**Figure legends**

Figure S1: Subgroup analysis on the impact of oral health education on the population's plaque index.

Figure S2: Subgroup analysis on the impact of oral health education on the population's gingival index.

Figure S3: Subgroup analysis on the impact of oral health education on the population's self-efficacy.

Figure S4: Subgroup analysis of the impact of oral health education on the frequency of brushing in the population.

Figure S5: Subgroup analysis on the impact of oral health education on the population's oral hygiene knowledge.

Figure S6: Subgroup analysis on the impact of oral health education on the population’s oral hygiene practice.

**Table S1: Search strategy on Pubmed.**

| #1 | Search “Health Education” [MeSH] |
| --- | --- |
| #2 | Search (Education, Health [All Fields]) OR (Community Health Education [All Fields]) OR (Education, Community Health [All Fields]) OR (Health Education, Community [All Fields]) |
| #3 | Search #1 OR #2 |
| #4 | Search “Periodontal Health” [All Fields] |
| #5 | Search “Randomized Controlled Trial” [All Fields] |
| #6 | Search #4 AND #5 |
| #7 | Search #3 AND #6 |

**Figure S1**


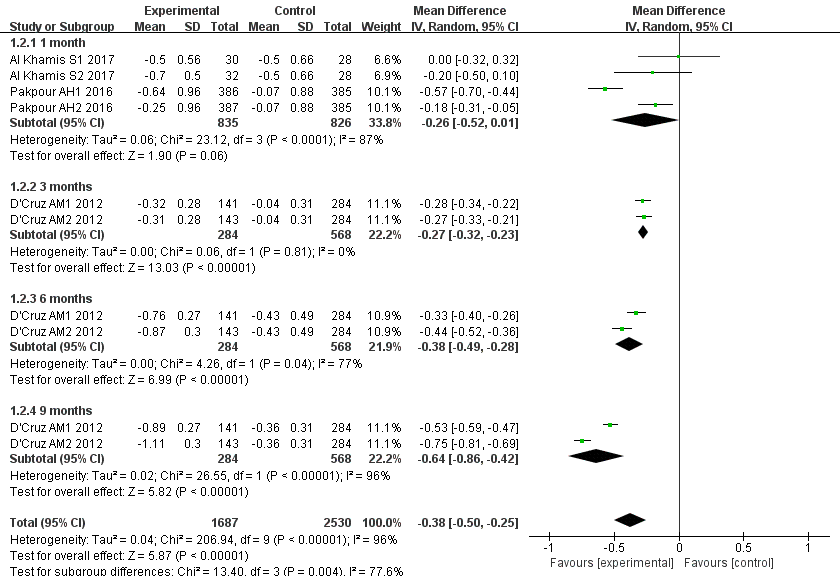


**Figure S2**


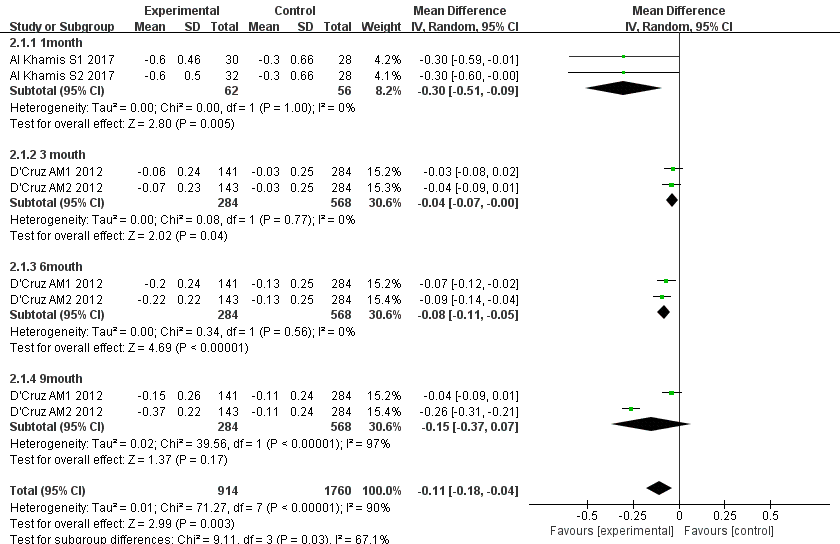


**Figure S3**


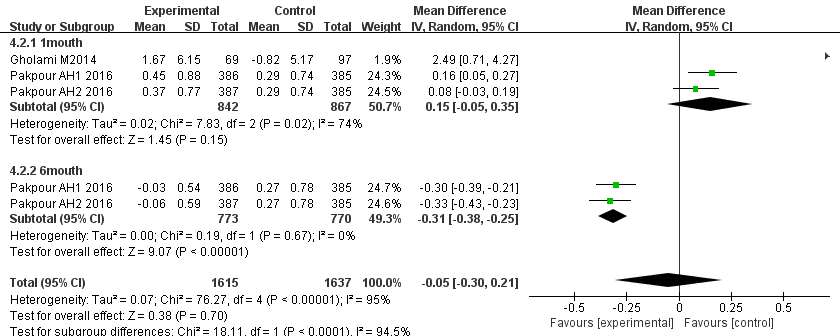


**Figure S4**


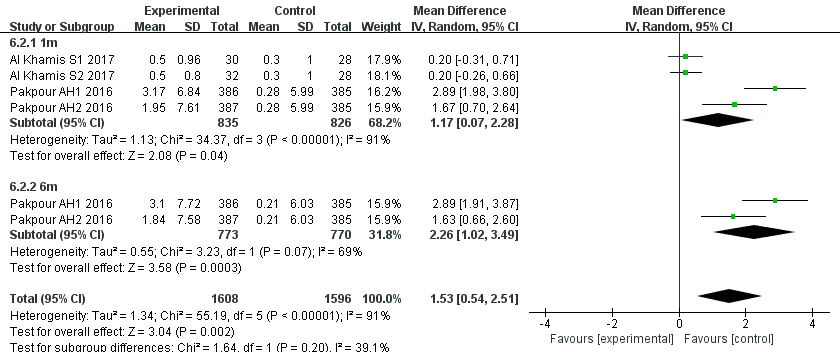


**Figure S5**


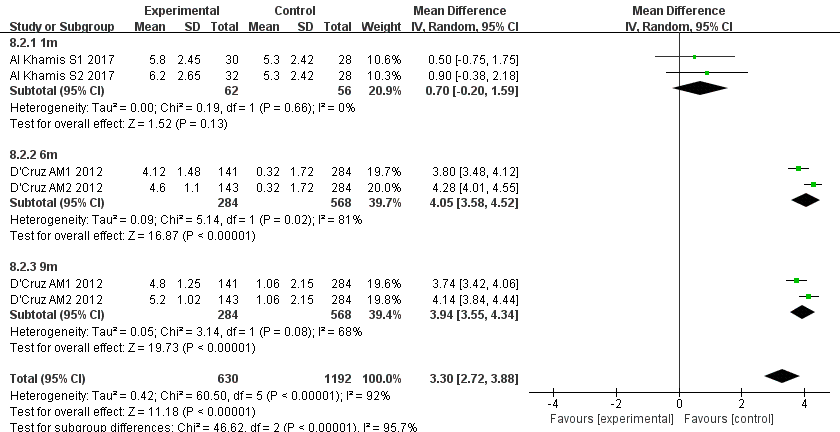


**Figure S6**


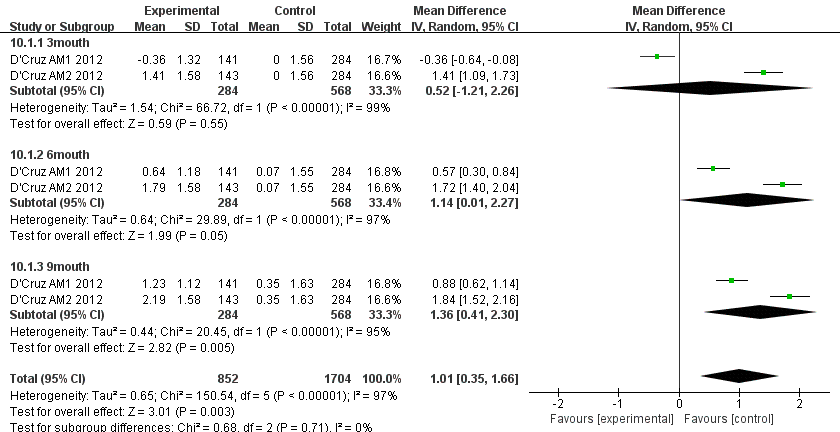

Supplement: Supplementary file 1 — Supporting Information Both Supporting materials can be found in their respective sections of the manuscript. Figure S1. Subgroup analysis on the impact of oral health education on the population’s plaque index. Figure S2. Subgroup analysis on the impact of oral health education on the population’s gingival index. Figure S3. Subgroup analysis on the impact of oral health education on the population’s self‐efficacy. Figure S4. Subgroup analysis of the impact of oral health education on the frequency of brushing in the population. Figure S5. Subgroup analysis on the impact of oral health education on the population’s oral hygiene knowledge. Figure S6. Subgroup analysis on the impact of oral health education on the population’s oral hygiene practice. Table S1. Search strategy on Pubmed. [file IJOD-2026-7955459-s001.docx]
